# Experimental Validation of Depth Cameras for the Parameterization of Functional Balance of Patients in Clinical Tests

**DOI:** 10.3390/s17020424

**Published:** 2017-02-22

**Authors:** Francisco-Ángel Moreno, José Antonio Merchán-Baeza, Manuel González-Sánchez, Javier González-Jiménez, Antonio I. Cuesta-Vargas

**Affiliations:** 1 MAPIR-UMA Group, Department Ingeniería de Sistemas y Automática. Universidad de Málaga, Instituto de Investigación Biomédica de Málaga (IBIMA), Málaga 29071, Spain; famoreno@uma.es (F.-Á.M.); javiergonzalez@uma.es (J.G.-J.); 2 Departamento de Fisioterapia, Universidad de Málaga, Grupo Clinimetría FE-14, Instituto de Investigación Biomédica de Málaga (IBIMA), Málaga 29071, Spain; jmerchan@uma.es (J.A.M.-B.); mgsa23@uma.es (M.G.-S.); 3 Departamento de Ciencias de la Salud, Universidad de Jaén, Jaén 23071, Spain; 4 School of Clinical Sciences of the Faculty of Health, the Queensland University of Technology, Brisbane QLD 4000, Australia

**Keywords:** depth camera, inertial sensor, multi-directional reach test, timed up and go, healthy young adults

## Abstract

In clinical practice, patients’ balance can be assessed using standard scales. Two of the most validated clinical tests for measuring balance are the Timed Up and Go (TUG) test and the MultiDirectional Reach Test (MDRT). Nowadays, inertial sensors (IS) are employed for kinematic analysis of functional tests in the clinical setting, and have become an alternative to expensive, 3D optical motion capture systems. In daily clinical practice, however, IS-based setups are yet cumbersome and inconvenient to apply. Current depth cameras have the potential for such application, presenting many advantages as, for instance, being portable, low-cost and minimally-invasive. This paper aims at experimentally validating to what extent this technology can substitute IS for the parameterization and kinematic analysis of the TUG and the MDRT tests. Twenty healthy young adults were recruited as participants to perform five different balance tests while kinematic data from their movements were measured by both a depth camera and an inertial sensor placed on their trunk. The reliability of the camera’s measurements is examined through the Interclass Correlation Coefficient (ICC), whilst the Pearson Correlation Coefficient (*r*) is computed to evaluate the correlation between both sensor’s measurements, revealing *excellent* reliability and *strong* correlations in most cases.

## 1. Introduction

When assessing balance of patients it is important to analyze the subject in static, semi-static and dynamic situations [1,2,3]. In clinical practice, balance is typically assessed by functional tests [1,2,3] that evaluate the motor reactions of the subject while performing a given standard functional movement [1,2,3]. These reactions are representative of the control of equilibrium, motor coordination and biomechanics by dependent sensory components of the vestibular and visual system [1,2,3]. The Timed Up and Go (TUG) test and the Multi-Directional Reach Test (MDRT) are two of the most validated functional tests for measuring balance in clinical practice [1,4,5].

Different instruments for kinematic analysis have been used to objectify the results of these functional tests [6,7,8,9,10,11,12,13]. Traditionally, the validation of instruments for kinematic analysis has been performed by using a 3D optical motion capture system [14]. However, the transfer of this method to clinical settings is limited due to its high cost, the space required for using it and the complexity of the integration and analysis of the recorded images/data [14,15]. For a kinematic analysis tool that is transferable to the clinical setting, the most important characteristics are: reliability, validity, portability, cost and ease of use [14,15,16,17]. In previous studies, inertial sensors have demonstrated excellent validity and reliability for kinematic analysis [9,10,11], hence being used as a reference for the validation of kinematic analysis instruments [18,19,20]. Unfortunately, the main disadvantages of using these devices are twofold: (1) the need for a wired connection to a computer, which can eventually limit the patient’s freedom of movement during the tests and (2) the need for the sensors need to be in direct contact with the patients’ body, which can be perceived as somehow invasive.

In recent years, depth cameras have been integrated into rehabilitation protocols in patients with different diseases, such as stroke survivors [13,21,22,23], Parkinson’s disease [24], and cerebral palsy [25], and in different movements such as jumping [26], walking [21,27,28], running [27] or even for transferring a person to a wheelchair [29]. Among the most similar works to our proposal, the paper series from Clark et al. [30,31] also presents the Kinect camera as a potential instrument for the assessment of standing balance and postural control. These works, though, do not cover neither all the four variants of the MDRT (specifically, the backwards MDRT), nor the TUG test. Furthermore, instead of inertial sensors, in these studies, a multi-camera 3D motion analysis system and a marker-based 3D motion analysis (3DMA) system have been employed, respectively, as the reference tool to evaluate the reliability and feasibility of the depth camera’s measurements. Our work focuses on evaluating both the reliability of depth cameras as tools for the kinematic analysis of the TUG and MDRT functional tests and their concurrent validity with respect to inertial sensors, hence complementing the above-mentioned works.

Finally, our implementation of the synchronized recording of data from both kinds of sensors has been added to a robotic software library that is freely available to the scientific community so that it can be easily employed by clinical professionals.

## 2. Materials and Methods

### 2.1. Design and Participants

In this work, the data obtained from an inertial sensor and a depth camera that evaluate a patient’s movement in a set of balance tests were compared. The inertial sensor measurements were taken as the reference to assess the performance of the depth camera in this task. For that, the Pearson Correlation Coefficient between the data gathered from both devices has been computed. The reliability of the camera data was evaluated through the Interclass Correlation Coefficient (ICC) and the Standard Error of Measurement (SEM), establishing the criterion-related validity of the inertial sensor.

The inclusion criteria for the participants were as follows: healthy young adults, able to remain standing for 60 s and to walk over 3 m without any external help. The exclusion criteria were: subjects presenting any limitations that could make it difficult for them to either stand or walk, or any inability to understand and follow the instructions required to perform the tests.

### 2.2. Ethical Principles

The participants received an information sheet that explained in detail the nature of the study. They were also able to contact the researchers with any questions they may have about the study. Besides, before starting the study, the participants signed a consent form to state their voluntary participation and consent to the transfer of the data collected during the measurements. Personal data were protected according to the Spanish Organic Law on Personal Data Protection 19/55. Moreover, the study was carried out in compliance with the guidance on Good Clinical Practice of the International Conference on Harmonization. This ensures the rights, safety and welfare of the participants in the study in accordance with the principles of the Declaration of Helsinki. The ethics certificate (FCCSS 11-11) was granted by the ethics committee of the Faculty of Health Sciences at the University of Málaga.

### 2.3. Measurement Instruments

#### 2.3.1. Inertial Sensor

**Figure 1:**
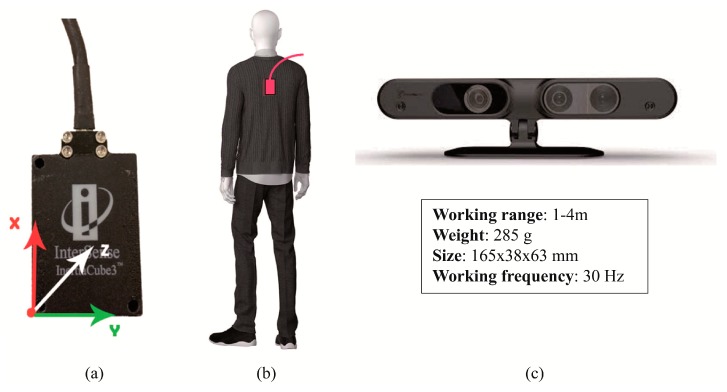
(**a**) Axes orientation of the inertial sensor and (**b**) its position on the participant’s body. (**c**) PrimeSense’s RGB-D camera and its main characteristics.

The InertiaCube3TM sensor from InterSense Inc. (Bedford, MA, USA) was used to measure inclination angles and angular rates. This device is a small inertial measurement unit that provides 3-Degree of Freedom (DOF) orientation data at a maximum update rate of 180 Hz with an accuracy of 1° in yaw angle and 0.25° in both pitch and roll. Its small size and weight (only 17 g) makes this device suitable for measuring body motion. The inertial sensor was placed within a Heart Rate Monitor chest strap, which was firmly attached to the patient so that the device was located as close as possible to the patient’s skin, between the scapulas (T7), as shown in Figure 1a,b. The measurements recorded by this sensor were employed as a benchmark to evaluate those obtained from the depth camera described below.

#### 2.3.2. Depth Camera

**Figure 2:**
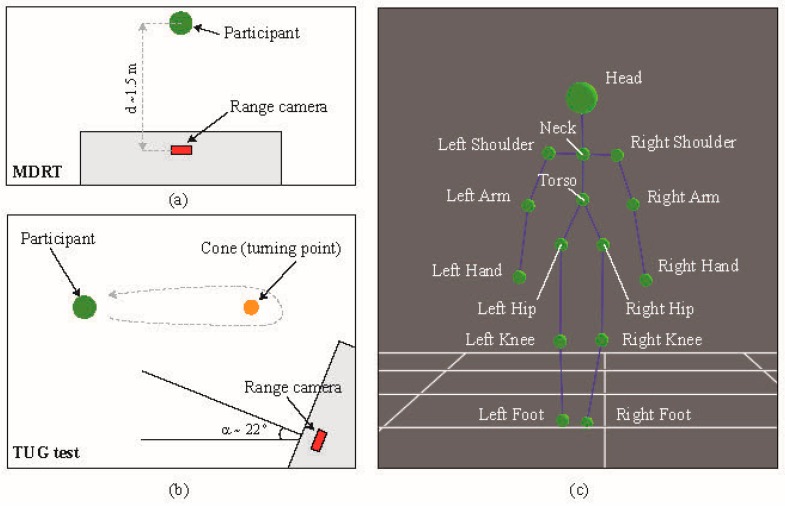
Experiment setup for the (**a**) MDRTs and the (**b**) TUG test. (**c**) Joints detected with the depth camera.

A depth camera (also known as range camera) manufactured by PrimeSense (Tel Aviv, Israel) was used in this study, whose main characteristics are displayed in Figure 1c. An extensive description of the physical and technological principles of depth cameras can be found elsewhere [32]; to summarize, there are two main types of depth camera: those based on time-of-flight (ToF) and those based on structured light (SL). The former operate by measuring the time of flight of a set of light signals emitted by the camera and bounced off the objects in the scene. The latter projects a specific pattern of structured infrared (IR) light onto the scene; this is then deformed by the 3D shape of the objects in it. This deformed pattern is captured by an IR camera that is part of the same device and compared with the original pattern in order to estimate the depth of the pixels in the captured image. In this study we used a SL-based camera. In order to take measurements for the MDRT, this device was placed with its optical axis perpendicular to the direction of the tested movements. The distance between the camera and the participant was set to approximately 1.5 m. However, for the TUG test, it was empirically found that placing the camera at a certain angle (around 22°) with respect to the direction of movement resulted in clearer measurements and reduced the possibility of losing track of the skeleton. These two experimental setups can be seen in Figure 2a,b.

#### 2.3.3. Data Recording and Processing

In order to record synchronized data from both sensors, their drivers were integrated into an open-source software library called MRPT [33], which allowed the use of the *rawlog-grabber* application (included in the library). This application provides concurrent access to a wide range of sensors usually employed in robotics, including inertial sensors and depth cameras, hence facilitating the simultaneous recording of time-stamped data from both devices. Subsequently, the depth information provided by the range camera is used to derive a high-level representation of the patient’s skeleton, composed of the 3D coordinates of a set of 15 joints (see Figure 2c). Both the depth and the skeleton information are obtained through the software libraries OpenNI2 and NiTE2, respectively. Finally, from the estimated position of the skeletal joints during the test, it is possible to compute the range-based parameterization of the patient’s movement (mainly inclination angles and angular speed), which was compared against the data provided by the inertial sensor. For the benefit of the scientific community, the software developed for this study has been publicly released as part of the above-mentioned robotic library [34].

Specifically, the 3D positions of the joints labelled Neck and Torso in Figure 2c were used to compute the angles between them for all tests. This corresponds to measuring the body motion at the T7 position, which would lie along the line between the above-mentioned joints. The inertial sensor was also placed at that location. The changes in the orientation of the inertial sensor, caused by movements in all three dimensions of space were measured as follows (Figure 1a): Flexion-Extension (*α*, pitch angle): rotation axis was *Y*, with positive data indicating flexion, and negative values indicating extension. Rotation (*β*, yaw angle): the rotation axis was *X*, where positive data indicated right rotation, while negative values indicated left rotation. Finally, inclination (*γ*, roll angle): the inclination axis was *Z*, where positive data indicated right inclination, while negative values indicated left inclination. For the case of the depth camera, let *P_N_ = (X_N_,Y_N_,Z_N_)* and *P_T_ = (X_T_,Y_T_,Z_T_)* be the 3D spatial coordinates of the Neck and Torso joints as measured by the range camera, respectively. Then, the equivalent Flexion-Extension (*α*) and Inclination (*γ*) angles can be computed as:

$$\alpha =arctan\left(\frac{X_{N}-X_{T}}{Y_{N}-Y_{T}}\right),\;\gamma =arctan\left(\frac{Z_{T}-Z_{N}}{Y_{N}-Y_{T}}\right)$$

Similarly, the Rotation angle (*β*) can be computed from the coordinates of the Left and Right Shoulders: *P_L_ = (X_L_,Y_L_,Z_L_)* and *P_R_ = (X_R_,Y_R_,Z_R_)*, respectively, through:

$$\beta =arctan\left(\frac{X_{R}-X_{L}}{Z_{L}-Z_{R}}\right)$$

**Figure 3:**
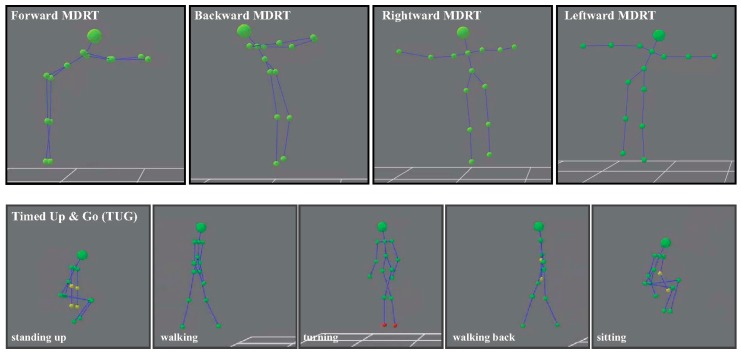
Skeleton’s movement detected in the MDRTs and the TUG test.

Figure 1a,b depict the orientation and fixation system of the inertial sensor. In turn, Figure 3 shows some examples of the skeleton’s movements in the MDRT and TUG tests as detected with the depth camera.

### 2.4. Functional Tests

#### 2.4.1. Timed Up and Go

The Timed Up and Go test is a quick and effective test that is widely used in clinical practice to evaluate the mobility and fall risk of patients [1,35,36]. In this test, the subject is initially seated on a chair without armrests [37]. Then, he or she is asked to get up and walk for 3 meters until reaching a turning point (a cone in the experiments performed here), turn around it, return to the chair and sit down again, all of this at a normal, natural speed for the participant. The subjects were allowed to practice this exercise before taking the measurements. Subsequently, three trials were recorded so that the one in which the subject stood up with the greatest acceleration was chosen as the best trial for this study [38]. The reliability data recorded by this dynamic balance and gait test in stroke patients are excellent (ICC > 0.96) [39].

#### 2.4.2. Multi-Directional Reach Test

The Multi-Directional Reach Test (MDRT) is a modification of the Functional Reach Test (FRT) employed to evaluate the static balance and the limitations in the patient’s anteroposterior and mediolateral stability, by measuring their range in four different directions: forward, backward, rightward and leftward [40,41,42]. The MDRT is widely considered as a simple screening tool for assessing balance, with high reliability (ICC = 0.942) and validity (concurrent validities of MDRT with TUG and Berg Balance Scale are *r* = [0.26, 0.44] and *r* = [0.36, 0.48], respectively) [40,42].

### 2.5. Outcome Variables

**Figure 4:**
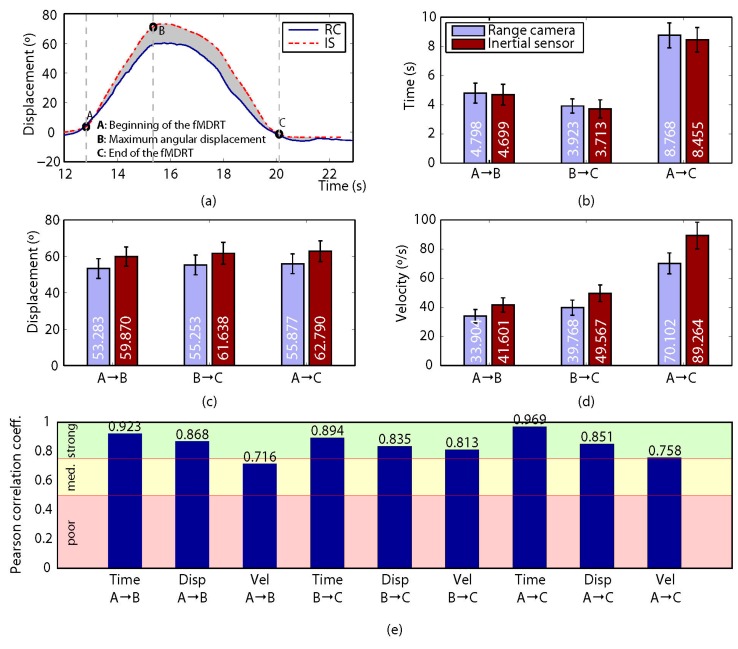
Results for the forward MDRT. (**a**) Comparison of the measured displacement provided by the depth camera (solid blue) and the inertial sensor (dashed red) for a representative participant. The positions of the defined control points are also shown; (**b**–**d**) mean time (with CI 95%), mean angular displacement (with CI 95%) and mean angular velocity (with CI 95%), respectively, over all the participants for the intervals defined by the control points; (**e**) Pearson Correlation Coefficient (r) (p < 0.005) between the devices for all the measured variables. (Time is expressed in seconds, angular displacement in degrees, and angular velocity in degrees per second).

**Figure 5:**
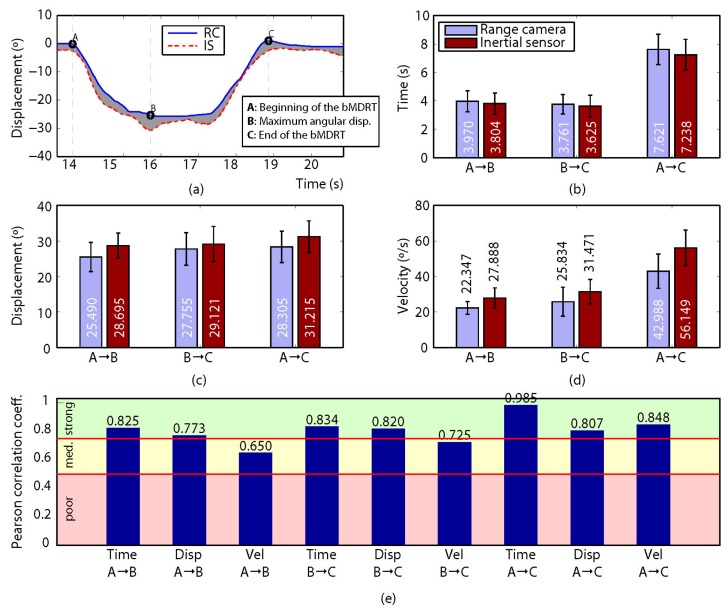
Results for the leftward MDRT. (**a**) Comparison of the measured displacement provided by the depth camera (solid blue) and the inertial sensor (dashed red) for a representative participant. The positions of the defined control points are also shown; (**b**–**d**) mean time (with CI 95%), mean angular displacement (with CI 95%) and mean angular velocity (with CI 95%), respectively, over all the participants for the intervals defined by the control points; (**e**) Pearson Correlation Coefficient (r) (p < 0.005) between the devices for all the measured variables. (Time is expressed in seconds, angular displacement in degrees, and angular velocity in degrees per second).

**Figure 6:**
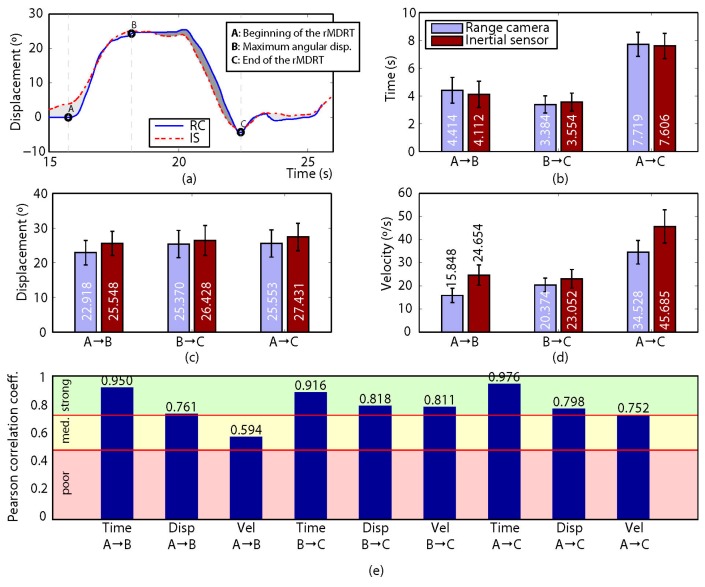
Results for the rightward MDRT. (**a**) Comparison of the measured displacement provided by the depth camera (solid blue) and the inertial sensor (dashed red) for a representative participant. The positions of the defined control points are also shown; (**b**–**d**) mean time (with CI 95%), mean angular displacement (with CI 95%) and mean angular velocity (with CI 95%), respectively, over all the participants for the intervals defined by the control points; (**e**) Pearson Correlation Coefficient (r) (p < 0.005) between the devices for all the measured variables. (Time is expressed in seconds, angular displacement in degrees, and angular velocity in degrees per second).

**Figure 7:**
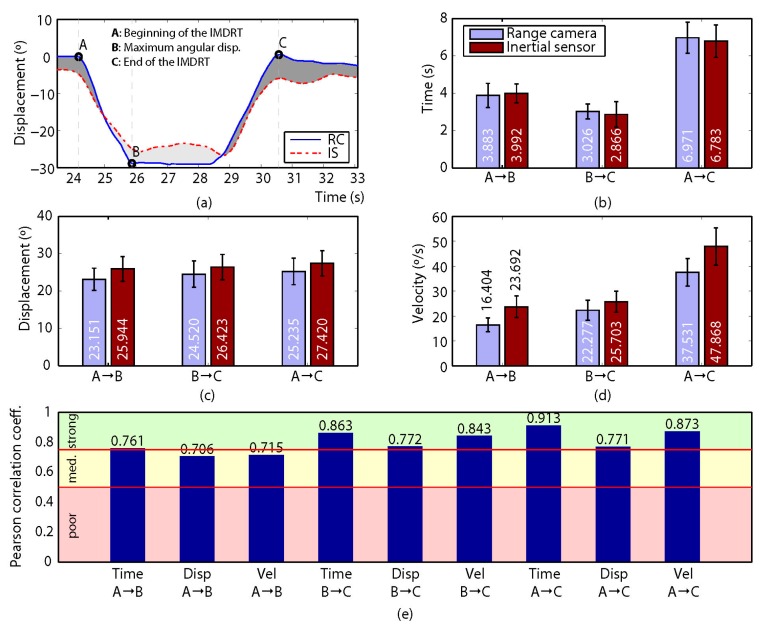
Results for the backward MDRT. (**a**) Comparison of the measured displacement provided by the depth camera (solid blue) and the inertial sensor (dashed red) for a representative participant. The positions of the defined control points are also shown; (**b**,**c**,**d**) mean time (with CI 95%), mean angular displacement (with CI 95%) and mean angular velocity (with CI 95%), respectively, over all the participants for the intervals defined by the control points; (**e**) Pearson Correlation Coefficient (r) (p < 0.005) between the devices for all the measured variables. (Time is expressed in seconds, angular displacement in degrees, and angular velocity in degrees per second).

**Figure 8:**
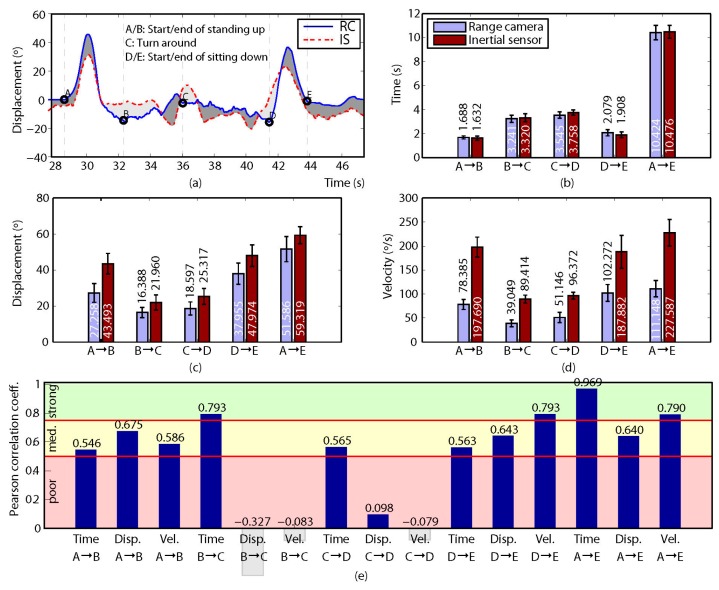
Results for the TUG test. (**a**) Comparison of the measured displacement provided by the depth camera (solid blue) and the inertial sensor (dashed red) for a representative participant. The positions of the defined control points are also shown; (**b**–**d**) mean time (with CI 95%), mean angular displacement (with CI 95%) and mean angular velocity (with CI 95%), respectively, over all the participants for the intervals defined by the control points; (**e**) Pearson Correlation Coefficient (r) (p < 0.005) between the devices for all the measured variables. (Time is expressed in seconds, angular displacement in degrees, and angular velocity in degrees per second).

Two direct variables (time and displacement) were obtained from the data recorded for each participant, and an indirect variable (velocity) was calculated. For this, a set of so-called control points was defined so that they divided the gathered measurements into a collection of intervals with relevant information about the participant’s motion. Then, both the direct and the indirect variables mentioned above were computed for each interval. For the MDRT and the TUG test, three (A, B and C) and five (A to E) control points were defined, respectively (refer to subfigure (a) in Figure 4, Figure 5, Figure 6, Figure 7 and Figure 8 for some examples of the control points’ position for each test).

The outcome variables were selected according to a systematic review of the kinematic analysis of ambulation using a depth camera as the measuring instrument [21]. All studies included in this systematic review analyzed temporal, spatial and velocity variables [21]. In addition, these spatiotemporal and kinematic variables have previously been used and suggested for the two tests included in the present study, i.e., TUG and MDRT [43].

#### 2.5.1. Direct Variables

The following variables were extracted from the kinematic data provided by the inertial sensor and the depth camera in the pitch angle, for the forward and backward MDRT, and the yaw angle for the leftward and rightward MDRT (note that the corresponding control points are specified, and a short name for the variable—within square brackets—is provided, and will be employed in Section 3): *Thoracic maximum angular displacement and associated time*: Maximum angular variation in the involved axis from the test’s starting point (A) until reaching the imbalance peak (point of maximum angular displacement) (B) [Disp. A→B] and the time spent in this interval [Time A→B]. *Thoracic maximum angular displacement in return to initial position and associated time*: Maximum angular variation in the involved axis from the peak of imbalance (B) until the initial position is reached (C) [Disp. B→C] and the time spent in this interval [Time B→C]. *Total MDRT thoracic maximum angular displacement and associated time*: Maximum angular variation for the whole MDRT test (from A to C) [Disp. A→C] and the time spent in this interval [Time A→C].

In the case of the TUG test the following direct variables were extracted for one degree of freedom (pitch angle): *Thoracic maximum angular displacement “sit-to-stand” and associated time*: Maximum angular variation from when the participant begins to stand up (A) until he or she is standing (B) [Disp. A→B] and the time spent on this movement [Time A→B]. *Thoracic maximum angular displacement until the turn and associated time*: Maximum angular variation between the patient’s first step (B) and reaching the turning point (C) [Disp. B→C] and the time spent on this movement [Time B→C]. *Thoracic maximum angular displacement “way-back” and associated time*: Maximum angular variation between reaching the turning point (C) and the point immediately before the participant is starting to sit down (D) [Disp. C→D] and the time spent on this movement [Time C→D]. *Thoracic maximum angular displacement “stand-to-sit” and associated time*: Maximum angular variation from when the participant is starting to sit down (D) until he or she is completely sat down (E) [Disp. D→E] and the time spent on this movement [Time D→E]. *Total TUG thoracic maximum angular displacement and associated time*: Maximum angular variation produced throughout the whole movement (from A to E) of the TUG test [Disp. A→E] and the time spent on it [Time A→E].

#### 2.5.2. Indirect Variables

From the data previously extracted in the MDRT, the following indirect variables were computed for each participant: *Thoracic maximum angular velocity*: Maximum angular velocity at which the participant performs from the start of the test (A) until reaching the imbalance peak (B) [Vel. A→B]. *Maximum angular velocity until return to initial position*: Maximum angular velocity at which the patient returns from the imbalance peak (B) to the starting position (C) [Vel. B→C]. *Total MDRT maximum angular velocity*: Maximum angular velocity at which the whole test is completed (from A to C) [Vel. A→C].

Finally, for the TUG test, the following indirect variables were calculated from the recorded data: *Maximum angular velocity “sit-to-stand”*: Maximum angular velocity at which the participant completely stands up (from A to B) [Vel. A→B]. *Maximum angular velocity until turn*: Maximum angular velocity at which the participant walks until the turning point (from B to C) [Vel. B→C]. *Maximum angular velocity “way-back”*: Maximum angular velocity of returning from the turning point to the chair (from C to D) [Vel. C→D]. *Maximum angular velocity “stand-to-sit”*: Maximum angular velocity at which the participant completely sits down (from D to E) [Vel. D→E]. *Total TUG maximum angular velocity*: Maximum angular velocity at which the participant completed the whole TUG test (from A to E) [Vel. A→E].

### 2.6. Procedure

First, the researchers briefly explained to the participants the nature of the tests so that they could sign an informed consent and their demographic data could be collected (i.e., gender, age, height and weight). After that, each of the experiments within the two groups of tests (MDRT and TUG) was explained to the participants, specifying that they were allowed to practice each one of them first. Next, the researchers placed the inertial sensor on the trunk (at the level of the T7) of the participant, and the depth camera in the appropriate position, as already explained in the previous section (please refer to Figure 1 and Figure 2). Then, the participants performed three repetitions of each test to ensure their reliability, hence allowing the researchers to choose the best trial for each experiment. The trial in which the patient achieved the largest range (measured with a standard measuring tape), and the largest acceleration [38] was selected as representative for the MDRT and TUG test, respectively. Finally, the researchers asked the participants to remain at a neutral, static position for three seconds at both the beginning and the end of the tests, in order to clearly identify the movement in the later analysis.

### 2.7. Data Analysis

A descriptive analysis was performed of each of the outcome variables, obtained from both the inertial sensor and the depth camera. For each variable and sensor, reliability values were calculated as a way of monitoring the measurements, by computing the Interclass Correlation Coefficient (ICC), following the model published by Shrout and Fleiss in [44], and the Standard Error of Measurement (SEM). Such reliability was classified as excellent (ICC ≥ 0.80), good (0.80 > ICC ≥ 0.60), moderate (0.60 > ICC ≥ 0.40) and poor (ICC < 0.40) [11]. In addition, the mean value of each variable based on measurements from all the participants was calculated, with confidence intervals (CI) of 95%. The Pearson Correlation Coefficient (*r*) between the measurements provided by the two devices was calculated. The resulting correlation values were classified into three categories: poor (*r* ≤ 0.49), moderate (0.50 ≤ *r* ≤ 0.74) and strong (*r* ≥ 0.75) [45]. The index of significance was set at or below *p* = 0.005. The Statistical Package for the Social Sciences (SPSS) (version 19.0 for Windows, SPSS Inc., Chicago, IL, USA) was employed in this statistical analysis.

## 3. Results

Twenty healthy young adults (*n* = 20, 12 females and 8 males) were recruited to participate in the present study. Participants presented an average age, height and weight of 20.6 years (±2.30), 1.71 m (±0.09) and 68.75 kg (±13.41), respectively, and lacked any limitations regarding standing, gait or understanding the instructions.

Table 1 presents the ICC and SEM values computed to evaluate the reliability of the measurements for the MDRT variants, for both devices and for each of the intervals into which the tests were divided. In the case of the MDRT (Table 1), the ICC parameter ranged from ICC = 0.897 ([Disp. B→C] (◦)) to ICC = 0.931 ([Time A→B] and [Time A→C] (s)) for the depth camera. For the inertial sensor, it ranged between ICC = 0.898 ([Disp. B→C] (◦)) and ICC = 0.931 ([Time A→B], [Time B→C] and [Time A→C] (s)).

For the TUG test, in turn, the reliability of the measurements made by both the depth camera and the inertial sensor is presented in Table 2, showing ICC values that ranged from ICC = 0.813 to ICC = 0.844, and from ICC = 0.807 to ICC = 0.853, respectively.

Regarding the correlation between the devices, Figure 4, Figure 5, Figure 6, Figure 7 and Figure 8 show the results obtained when comparing the measurements for the four variants of the MDRT and the TUG test. Each one of these figures contains five sub-figures with the following information: (a) an example of the displacement measurement provided by both the depth camera (in solid blue) and the inertial sensor (in dashed red) for a representative participant. The positions of the defined control points are also depicted in the figure. (b) Mean time (with CI 95%) over all the participants for the intervals defined by the control points. This corresponds to variables [Time X→Y] described in the previous section. (c) Mean angular displacement (with CI 95%) over all the participants for the intervals defined by the control points. This corresponds to variables [Disp. X→Y] described in the previous section. (d) Mean angular velocity (with CI 95%) over all the participants for the intervals defined by the control points. This corresponds to variables [Vel. X→Y] described in the previous section. (e) Pearson Correlation Coefficient (*r*) (with significance p < 0.005) between the devices for all the measured variables (i.e., 9 for the MDRT and 15 for the TUG test).

For the MDRT, the data obtained from the depth camera was strongly correlated with that from the inertial sensor in most of the evaluated variables and for all the test’s variants. As an example, the correlation between the devices for the forward MDRT ranged from *r* = 0.716 ([Vel. A→B]) to *r* = 0.969 ([Time A→C]). For the TUG test, however, the correlations were lower due to limitations in the operation of the depth camera, as will be discussed later. Nevertheless, some of the variables still showed high levels of correlation.

## 4. Discussion

The aim of this study was to validate the depth camera as a device for parameterization and kinematic analysis of functional tests of balance. As stated above, the reliability of the measurements provided by both devices was analyzed by computing the Interclass Correlation Coefficients (ICC) and the Standard Error of Measurement (SEM); these demonstrated *excellent* reliability, and are presented individually for each balance test.

On the other hand, by analyzing and comparing the results of the Pearson Correlation Coefficients (*r*) in all four MDRT variants and the TUG test, it can be seen that such correlations ranged from moderate (with a minimum of *r* = 0.546 for [Time A→B] in the TUG test) to strong (with a maximum of *r* = 0.985 for [Time A→C] in the backward MDRT), except for the angular displacement and velocity variables in the walking stage of the TUG test (i.e., from control points B to D).

For the four variants of MDRT, the analysis of the *r* values between the depth camera and the inertial sensor for the considered intervals revealed that more than 80% of the variables presented a *strong* correlation, with the rest having a moderate correlation. In general, worse correlation values were found for the angular velocity variables in each interval, with the worst case for the MDRT in the rightward MDRT [Vel. A→B] variable, with *r* = 0.594, while the values in the same interval for [Time A→B] and [Disp. A→B] were *r* = 0.950 and *r* = 0.761 respectively. This may be mainly because, for the depth camera, they were calculated indirectly from the angle values, which, in turn, were estimated from the measurements of the joints’ 3D positions. Initial errors of these measurements are propagated along a series of linear and nonlinear functions employed to compute the angular velocity [43]. Although such measurements are filtered to eliminate high frequency noise as far as possible, the propagated errors become larger when estimating velocity variables, thus reducing the accuracy.

Overall, the results on both the reliability and validity of the depth camera reveal it as a suitable alternative to inertial sensors in order to assess functional balance tests, hence fulfilling the objective of this study.

### 4.1. Forward MDRT

The reliability of the measurements made by the depth camera over time, angular displacement and angular velocity in the three intervals of the forward MDRT fell between ICC = 0.897 for [Disp. B→C] and ICC = 0.913 for [Time A→B], these being considered excellent values of reliability (please refer to Figure 4). The inertial sensor also showed excellent values in the three intervals for each variable, ranging from ICC = 0.898 for [Disp. B→C] to ICC = 0.914 for [Time A→B].

By observing the correlations between the depth camera and the inertial sensor for time, displacement and velocity in the three defined intervals in the forward MDRT, it can be seen that eight of the nine variables showed a strong correlation, while for the velocity A→B the correlation was moderate. The slight decrease in the velocity A→B correlation may be due to the propagation of uncertainty from measurements of joints to the final estimate of the angular velocity [46]. This study is the first to compute the correlation in the measurement of functional tests of balance between a depth camera and an inertial sensor. However, previous studies have conducted correlation analysis in balance and gait tests between a depth camera and a 3D optical motion capture system, obtaining similar results. For example, Clark et al. [30,31] analyzed the anteroposterior displacement of the FRT measured using a depth camera and compared it with a multiple-camera 3D motion analysis system and a marker-based 3D motion analysis system, respectively, obtaining a *strong* correlation between methods (*r* = 0.93 and *r* = 0.89). These results are consistent with the correlations obtained in this paper for the angular displacement (*r* = 0.86 in interval A→B, *r* = 0.83 in interval B→C and *r* = 0.85 in interval A→C). Other studies, though, have reported lower correlations for the anteroposterior displacement (*r* = 0.30 and *r* = 0.59) [24,47].

### 4.2. Backward MDRT

*Excellent* reliability values were also found in the backward MDRT for the depth camera, as shown in Figure 5, with a minimum value of ICC = 0.915 for [Vel. A→C] and a maximum of ICC = 0.931 for [Time A→B] and [Time A→C]. Reliability values for the inertial sensor were also found to be excellent, ranging from ICC = 0.918 ([Disp. B→C] and [Vel. B→C]) to ICC = 0.931 for the Time variables in the three intervals.

In terms of validity, the results obtained for the variables measured in the backward MDRT show that seven of the nine measured variables presented a strong correlation, while there was a moderate correlation for velocity in intervals A→B and B→C. Again, the propagation of the measurement uncertainty of joint positions resulted in an increase in the error in the estimation of these variables [46]. These results could not be compared with other correlations as no previous studies have compared a depth camera with another parameterization system for the backward MDRT.

### 4.3. Rightward and Leftward MDRT

The ICC values computed from the depth camera for the rightward MDRT (between ICC = 0.902 for [Vel. A→C], and ICC = 0.922 for [Time B→C] and [Time A→C]) and leftward MDRT (in the range between ICC = 0.910 for [Vel. A→C], and ICC = 0.927 for [Time A→B] and [Time A→C]) were also excellent, as can be seen in Figure 6 and Figure 7, respectively. The same was true for the inertial sensor: rightward MDRT (from ICC = 0.905 for [Disp. B→C] and [Disp. A→C], to ICC = 0.922 for [Time B→C]) and leftward MDRT ranging from ICC = 0.912 for [Vel. B→C] to ICC = 0.927 for the Time variables in the three intervals.

The reliability of the measurements of the depth camera in all four variants of MDRT was consistent with that achieved by a depth camera in the study by Galna et al. [24] when compared with a high-frequency camera system in the parameterization of front (ICC = 0.978) and lateral reach (ICC = 0.957). Similarly, the reliability values found for the inertial sensor were consistent with those obtained using a similar device located on T7 in the parameterization of the FRT (from ICC = 0.829 to ICC = 0.878) [11].

With regards the correlation values obtained in the rightward MDRT, it can be seen that eight of the nine variables measured showed strong correlations, except for the velocity in interval A→B, which showed a moderate correlation (*r* = 0.59). This variable again showed the effect of the propagation of the errors of the original measurements [46]. In turn, for the leftward MDRT, the three variables in intervals A→C and B→C, along with the time in interval A→B, presented strong levels of correlation. The remaining two variables, on the other hand, showed moderate levels of correlation: displacement and velocity in interval A→B (*r* = 0.70) and (*r* = 0.71), respectively. The correlation values obtained for the displacement in the leftward (*r* = 0.70 A→B, *r* = 0.77 B→C and *r* = 0.77 A→C) and rightward MDRT (*r* = 0.76 A→B, *r* = 0.81 B→C and *r* = 0.80 A→C) in this work are consistent with the correlations of *r* = 0.86, *r* = 0.61, *r* = 0.92 and *r* = 0.87 reported by Galna et al. [24], Lim et al. [47], and Clark et al. [30,31], who compared measurements made by a depth camera and a 3D optical motion capture system for the lateral reach test.

### 4.4. Timed Up and Go

The depth camera also showed excellent measurement reliability for parameterizing the five intervals of the TUG (refer to Figure 8), with values between ICC = 0.813 for [Vel. A→E] and ICC = 0.844 for [Disp. C→D]. The values achieved for the angular displacement in interval A→B (“sit-to-stand”) (ICC = 0.835) and for the angular displacement in intervals C→D and B→C (“gait”) were consistent with the values ICC = 0.961 and ICC = 0.968 observed for the sit-to-stand phase and gait, respectively, in a similar study [24].

The reliability of the measurement of the inertial sensor was also excellent in the data gathered for the variables of the five intervals into which the TUG was divided (from ICC = 0.807 to ICC = 0.853). These values were consistent with those obtained in a study in which this test was parameterized with an inertial sensor (from ICC = 0.819 to ICC = 0.987) [11].

The analysis of the correlation of the three variables for each one of the five intervals in which the TUG was divided revealed values ranging from *r* = −0.327 to *r* = 0.969 (from poor to strong). In this particular test, limitations in the operating range of the camera resulted in noisier estimates than in previous tests. Specifically, both the start and the end of the movement occurred near the limit of the camera’s operative range, where the measurement position is less accurate. In addition, working so close to the limit may produce exceptional monitoring loss of one of the joints, affecting the final measurement [48,49,50]. This results in errors when determining the points defining the movement, especially for the B and D points, producing a significantly low correlation in the central sections of the movement.

The correlation in the angular displacement A→E (total displacement of the TUG test) and in the displacement A→B (“sit-to-stand”) found in this study was moderate (*r* = 0.64 and *r* = 0.67, respectively), while in the study by Galna et al. [24] both variables were strongly correlated (*r* = 0.82 and *r* = 0.99). In turn, the average velocity of the whole test showed a strong correlation (*r* = 0.79), consistent with the correlation reported by Mentiplay et al. (*r* = 0.99) [28], who measured the TUG test with a depth camera and a 3D optical motion capture system.

### 4.5. Limits and Strengths of the Study

The depth camera was unable to record clear data on the subject’s movement during the performance of the TUG due to range limitations. The three-meter path covered by the TUG coincides approximately with the limit of the depth camera’s operating range and so the sensor measurements became more erratic. It is likely, though, that these technological problems will be overcome in new camera models. The Kinect v2 camera, which is based on the ToF principle, has a greater field of view, which extends its ranging operation; however it is approximately twice as expensive as the camera used in this study and has to be powered externally rather than through USB. Another way of mitigating the impact of range limitations would be to use multiple devices to cover a larger area; however, this might cause other problems, such as interferences between devices, synchronization or extrinsic calibration of the cameras. These issues are beyond the scope of this work.

As a follow-up to this study, which was intended to validate the use of the depth camera as a valid measurement system in the parameterization of functional tests, other studies on populations with limited balance, such as elderly people and people with certain illnesses, will be carried out. In addition, a thorough analysis of the extracted kinematic data derived from the depth camera might reveal, for example, new indicators of risk of falling that could be used in daily clinical practice to make it easier to categorize elderly people among pre-frail, frail or on risk of fall.

On the other hand, this is one of the first studies to assess and parameterize devices that can be easily transferred from the lab to a clinical environment, hence minimizing the subjective impact that the balance tests presents.

## 5. Conclusions

This paper aimed at experimentally evaluating depth cameras as reliable instruments to perform kinematic analysis of balance tests and also as valid alternatives to inertial sensors, which are cumbersome, invasive and more expensive. Specifically, four variants of the Multi-Directional Reach Test (MDRT) and the Timed Up and Go (TUG) test were considered, as they represent two of the most validated functional tests for measuring balance in clinical practice. For this evaluation, a set of twenty healthy young adults were recruited and their motions were measured with both kinds of sensors while performing the tests.

The comparison and analysis of the results obtained in this study revealed that the depth camera is a reliable instrument to parameterize both the Multi-Directional Reach Test (MDRT) and the Timed Up and Go (TUG) test, yielding excellent values for the Interclass Correlation Coefficient (ICC) test. Furthermore, in the case of the MDRT, it also presented strong Pearson Correlation Coefficient values when compared with the criterion-related validity (inertial sensor) in most of the evaluated tests. However, the results also showed particular issues when employing depth cameras for the TUG test due to limitations in its operative range. These limitations could be mitigated, however, by using devices with a wider field of view or employing multi-camera setups. In any case, the capability of the depth camera to provide similar results than those obtained by the inertial sensor has been proved for most of the performed tests.

Finally, and as a contribution to the scientific community, the implementation of the synchronized recording of data from both sensors has been integrated into an open-source robotic software library that is freely available.

## 6. Ethics Approval and Consent to Participate

Ethical approval: All procedures performed in the study involving human participants were in accordance with the ethical standards of the institutional and/or national research committee and with the 1964 Helsinki Declaration and its later amendments. Informed consent: Informed consent was obtained from all participants included in the study. The ethics certificate (FCCSS 11-11) was granted by the ethics committee of the Faculty of Health Sciences at the University of Málaga

## Figures and Tables

**Table 1:** Reliability of the measure for all variants of MDRT.

		Forward MDRT		Backward MDRT		Rightward MDRT		Leftward MDRT	
		ICC	SEM	ICC	SEM	ICC	SEM	ICC	SEM
Time A→B	IS	0.914	0.335	0.931	0.361	0.920	0.456	0.927	0.243
	DC	0.913	0.327	0.931	0.350	0.921	0.441	0.927	0.308
Disp. A→B	IS	0.900	2.535	0.921	1.684	0.906	1.657	0.913	1.582
	DC	0.899	2.184	0.919	1.948	0.906	1.687	0.916	1.417
Vel. A→B	IS	0.904	2.360	0.921	2.728	0.915	2.099	0.920	2.079
	DC	0.905	2.184	0.920	1.697	0.910	1.471	0.920	1.320
Time B→C	IS	0.911	0.299	0.931	0.362	0.922	0.312	0.927	0.326
	DC	0.912	0.233	0.930	0.330	0.922	0.293	0.926	0.193
Disp. B→C	IS	0.898	2.852	0.918	2.359	0.905	2.053	0.913	1.614
	DC	0.897	2.580	0.917	2.192	0.906	1.882	0.913	1.695
Vel. B→C	IS	0.904	2.723	0.918	3.251	0.906	1.919	0.912	2.009
	DC	0.899	2.482	0.919	3.902	0.908	1.435	0.912	1.934
Time A→C	IS	0.912	0.401	0.931	0.519	0.921	0.434	0.927	0.415
	DC	0.912	0.409	0.931	0.508	0.922	0.415	0.927	0.396
Disp. A→C	IS	0.898	2.702	0.919	2.117	0.905	1.889	0.913	1.624
	DC	0.898	2.571	0.917	2.120	0.906	1.868	0.914	1.688
Vel. A→C	IS	0.907	4.347	0.922	4.759	0.916	3.437	0.922	3.578
	DC	0.900	3.443	0.915	4.597	0.902	2.418	0.910	2.630

IS: Inertial Sensor; DC: Depth Camera; ICC: Interclass Correlation Coefficient; SEM: Standard Error of Measurement.

**Table 2:** Reliability of the measure for the TUG test.

		TUG				TUG	
		ICC	SEM			ICC	SEM
Time A→B	Inertial Sensor	0.843	0.083	Time B→C	Inertial Sensor	0.843	0.158
	Depth Camera	0.843	0.061		Depth Camera	0.843	0.145
Disp. A→B	Inertial Sensor	0.835	2.736	Disp. B→C	Inertial Sensor	0.849	2.007
	Depth Camera	0.835	2.477		Depth Camera	0.841	1.374
Vel. A→B	Inertial Sensor	0.833	9.961	Vel. B→C	Inertial Sensor	0.853	3.939
	Depth Camera	0.827	5.048		Depth Camera	0.835	3.293
Time C→D	Inertial Sensor	0.843	0.106	Time D→E	Inertial Sensor	0.843	0.110
	Depth Camera	0.843	0.131		Depth Camera	0.843	0.123
Disp. C→D	Inertial Sensor	0.846	2.100	Disp. D→E	Inertial Sensor	0.831	2.849
	Depth Camera	0.844	1.740		Depth Camera	0.833	2.845
Vel. C→D	Inertial Sensor	0.839	3.393	Vel. D→E	Inertial Sensor	0.829	16.321
	Depth Camera	0.835	5.062		Depth Camera	0.818	8.417
Time A→E	Inertial Sensor	0.843	0.254				
	Depth Camera	0.843	0.285				
Disp. A→E	Inertial Sensor	0.834	13.201				
	Depth Camera	0.831	8.172				
Vel. A→E	Inertial Sensor	0.807	2.286				
	Depth Camera	0.813	3.347				
ICC: Interclass Correlation Coefficient SEM: Standard Error of Measurement							

## Supplementary Materials

All data are available from first and corresponding author. Also all data is under request in the institutional repository of the corresponding author. http://riuma.uma.es/xmlui.
